# Inaugural editorial

**DOI:** 10.1007/s44194-022-00004-z

**Published:** 2022-05-26

**Authors:** Songlin Wang, Alfred Yung

**Affiliations:** 1 Beijing Laboratory of Oral Health, Capital Medical University, Beijing, China; 2 The Universiy of Texas MD Anderson Cancer Center, Houston, USA

We write this inaugural editorial for the first issue of *Current Medicine* with great humility and honor. *Current Medicine* was launched in the post-COVID-19 era in a world devastated by unprecedented calamities that have destabilized economies and institutions. This pandemic must be rigorously examined by the international community to effectively build solutions that meet the upcoming challenges in a world transformed by this generational event. *Current Medicine* provides a suitable and relevant scientific platform to develop innovative science and technology for dissemination to a global community of healthcare partners.

Original clinical research of interest in clinical and basic medicine will be published in *Current Medicine* (*CM*), which is the official journal of National Institute for Data Science in Health and Medicine, Capital Medical University. Springer Nature, one of the most influential and biggest publishing houses in the area of medicine, is the publisher of *CM*. Led by an outstanding Editorial Board of international experts including academicians of Chinese Academy of Sciences or Engineering (Prof. Songlin Wang and Prof. Hongbing Shen), well-known Prof. Alfred Yung, Prof. Wenbin Li, and more than 40 prominent experts along with the scientific advisor of the Nobelist (Prof. Edvard I. Moser), this multidisciplinary open-access journal aims to lead the field in disseminating and exchanging significant scientific knowledge and discoveries worldwide. It offers an opportunity for clinicians, researchers, academics and the public to develop research agendas. It strives to be an influential journal with great international reputation by clinicians and researchers.

*CM *lays emphasis on scientific advances with new therapies or diagnostic tools, including pre-clinical research on innovative treatment and clinical trials, especially for precision medicine and discussions on clinical guidelines including expert consensus for various specialized diseases. *CM* publishes studies performed by multi-center groups in various disciplines of medicine, including clinical trials and cohort studies from large patient populations. These include head and neck diseases, basic and clinical research for cancers, neuroscience and clinical neurology, phase I/phase II/phase III clinical trials and studies for new drugs or therapies, as well as reviews oriented to the practicing internist and diagnostic puzzles.

To actively promote the dissemination of work published in the *CM*, an expedited review process is adopted*.* Articles will be published on the Journal’s web page (https://www.springer.com/journal/44194) immediately upon acceptance and accessed with no charge. To make the work published in *CM* more widely spread to international scientists, *CM* adopts the fully OA publishing mode. The Institute will sponsor the Article Processing Charge (APC), so no fee will be levied on the authors of *CM* for the first three years. Moreover, high-quality peer-review will be appreciated by our journal.

Human history is shaped by the innovations in medicine essential in treatment of novel diseases. To safeguard the survival of the species, it is necessary to continually cultivate the knowledge and technology supporting therapy and prevention. *CM* will support establishing new technologies and empowering medical professionals with high quality research for better care of their patients. *CM* is fortunate to receive the support from an excellent editorial team with an outstanding research and editorial experience in the field of both medicine and modern medical technology.

All in all, we look forward to working with all those who are or will be involved with the *CM*, including the readers, authors, associate editors, editorial board members, reviewers, and publisher’s team with the purpose of fulfilling the mission of *CM*.

